# Role of TRPV3 in immune response to development of dermatitis

**DOI:** 10.1186/1476-9255-6-17

**Published:** 2009-05-25

**Authors:** Kinichi Imura, Takeshi Yoshioka, Tsutomu Hirasawa, Tsuneaki Sakata

**Affiliations:** 1 Discovery Research Laboratories, Shionogi & Co, Ltd, 3-1-1 Futaba-cho, Toyonaka, Osaka 561-0825, Japan

## Abstract

**Background:**

Recently, it has been reported that the Gly573Ser substitution of transient receptor potential V3 (TRPV3) leads to increased ion-channel activity in keratinocytes. Our previous studies have indicated that the spontaneous hairless and dermatitis phenotypes of DS-*Nh *mice, which were newly established as an animal model of atopic dermatitis (AD), are caused by TRPV3^Gly573Ser^. Although this substitution causes hairlessness in several kinds of rodents, in our investigations, dermatitis developed in only a few animals. Here, we generated NC/Nga-*Nh *mice to elucidate the role of TRPV3^Gly573Ser ^in NC/Nga mice, which is one of the most studied animal models of AD.

**Methods:**

To establish and validate the new AD animal model, NC/Nga-*Nh *mice were generated using NC/Nga and DS-*Nh *mice, and their clinical features were compared. Next, T-cell receptor (TCR) Vβ usage in splenocytes, evaluation of bacterial colonization, and serological and histological analyses were carried out. Finally, repeated-hapten-application dermatitis was induced in these mice.

**Results:**

NC/Nga-*Nh *mice did not develop spontaneous dermatitis, whereas DS-*Nh *mice displayed this phenotype when maintained under the same conditions. Serological analysis indicated that there really was a phenotypic difference between these mice, and TCR repertoire analysis indicated that TCRVβ haplotypes played an important role in the development of dermatitis. Artificial dermatitis developed in DS and NC/Nga-*Nh *mice, but not in DS-*Nh *and NC/Nga mice. Histological and serological analyses indicated that mouse strains were listed in descending order of number of skin mast cells: DS-*Nh *> DS ≈ NC/Nga-*Nh *> NC/Nga, and serum IgE levels were increased after 2,4,6 trinitrochlorobenzene application in these mice. Serum IgE level in DS-*Nh *mice was lower than that mesured in other strains.

**Conclusion:**

Our results confirm the contribution of the TRPV3^Gly573Ser ^gene to the development of repeated hapten dermatitis, but not spontaneous dermatitis in NC/Nga mice.

## Background

Transient receptor potential (TRP) channels are expressed in almost all organs in the body and are thought to play important roles in maintaining vital functions [1]. They are key players in sensory systems and respond to temperature, touch, pain, osmolarity, pheromones, taste and other stimuli [2]. TRP channels can be divided into six main subfamilies: TRPA, TRPC, TRPM, TRPML, TRPP and TRPV [2]. Although TRPV3 is expressed in the skin, keratinocytes and hair follicles [3], and is activated by temperatures higher than 32–39°C, 2-aminoethoxydiphenyl borate (2-APB) and camphor [4], its detailed functions in animals have not been elucidated. We have reported previously that Gly573Ser substitution of TRPV3 is a gain-of-function mutation, as shown by the results of Ca^2+ ^influx stimulated by temperature [5,6]. Xiao *et al*. have speculated recently that Gly573Ser-substituted TRPV3 is active constitutively *in vivo *under normal physiological conditions [7].

In 1976, DS-*Nh *mice were artificially selected on the basis of hairless phenotype from a colony of an inbred DS strain, which was developed from outbred ddN stock obtained in 1954 from the Central Institute for Experimental Animals, Tokyo, Japan. DS-*Nh *mice also develop a form of dermatitis and are considered to be a model of human atopic dermatitis (AD), with the following features: (i) superantigen-producing *Staphylococcus aureus *is one of the causes of dermatitis; (ii) significantly increased serum levels of IgE, interleukin (IL)-4 and IL-13; (iii) increased numbers of whole mast cells and CD4-bearing T cells; (iv) increased serum and tissue levels of nerve growth factor (NGF); and (v) itching behavior becomes significantly severe [8-11]. Recently, we have reported that Gly573Ser substitution in TRPV3 causes spontaneous hairless and dermatitis phenotypes in DS-*Nh *mice [5,6,12,13]. Interestingly, according to our investigations, this substitution causes hairlessness in several species of rodents; however, at least one of these does not develop the dermatitis phenotype seen in DS-*Nh *mice [13].

NC/Nga mice were established in 1957 as an inbred strain by Kondo *et al*. [14]. NC/Nga mice develop AD-like dermatitis when they are kept under conventional conditions [15]. This spontaneous dermatitis is thought to be caused by house dust mites rather than *S. aureus*, because NC/Nga mice do not develop dermatitis when they are kept under the same conditions in which DS-*Nh *mice develop dermatitis (unpublished data). However, Hashimoto *et al*. have reported that development of eczema is closely related to the number of *S. aureus *on the skin of NC/Nga mice [16].

Here, we bred NC/Nga-*Nh *congenic mice to test the ability of the mutated TRPV3 to cause AD-like dermatitis in an NC/Nga background.

## Methods

### Animals

The NC/Nga-*Nh *congenic strain was established at Shionogi Aburahi Laboratories. Since the *Nh *non-hair phenotype is inherited in autosomal dominant mode, it was easy for us to introduce this phenotype to another strain of mouse using ordinary breeding methods. We segregated these strains according to non-hair phenotype. The congenic mice used in this study had undergone more than 10 generations. DS, DS-*Nh*, NC/Nga and NC/Nga-*Nh *mice were maintained in micro-isolator cages, exposed to a 12-h light/12-h dark cycle, and provided with standard feed and water *ad libitum*. Animals were housed in rooms, under specific pathogen-free (SPF) conditions for 5 weeks, and then moved to conventional conditions, or kept under SPF conditions. This study was conducted according to the guidelines for animal experimentation at Shionogi.

### Histological study

Paraffin sections were prepared from skin lesions for histochemical analysis. Hematoxylin and eosin staining was used for histopathological analysis to evaluate cellular infiltration, including mast cells, and hyperkeratosis.

### Evaluation of scratching and rubbing behavior

Scratching and rubbing behavior was evaluated according to itching score, as follows. We measured total time of scratching and rubbing by forepaws for 30 min. Scores: 0, total time ≤ 100 s; 1, 101–200 s; 2, 201–300 s; 3, 301–400 s; 4, 401–500 s; 5, 501–600 s; 6, 601–700 s; 7, 701–800 s; 8, 801–900 s; 9, 901–1000 s; and 10, > 1000 s.

### Serological analysis

Sera were collected from DS-*Nh *and NC/Nga-*Nh *mice at 20 weeks of age kept under SPF or conventional conditions, and stored at -80°C until use. The following cytokines were measured using Bio-Plex (Bio-Rad Laboratories, CA, USA) and ELISA kits (Biosource International, Camarillo, CA, USA and MBL, Nagoya, Japan) according to the manufacturers' instructions: IL-12(p40), IL-12(p70), IL-13, IL-17, eotaxin, granulocyte colony-stimulating factor (G-CSF), granulocyte-macrophage colony-stimulating factor (GM-CSF), interferon-γ (IFN-γ), keratinocyte-derived chemokine (KC), monocyte chemotactic protein-1 (MCP-1), macrophage inflammatory protein-1α (MIP-1α), MIP-1β, RANTES (regulated on activation, normal, T-cell-expressed and secreted) and tumor necrosis factor-α (TNF-α), IL-1α, IL-1β, IL-2, IL-3, IL-4, IL-5, IL-6, IL-9, IL-10, and IL-18.

### Analysis of mRNA for T-cell receptor (TCR) usage

Murine spleen cells (5 × 10^5^/well) were incubated in 96-well microplates (Corning Corster Co., Cambridge, MA, USA) in RPMI 1640, 10% FCS, 100 U/ml penicillin, and 100 mg/ml streptomycin, with or without 1 μg/ml staphylococcal enterotoxin C (SEC), at 37°C for 4 days. Crude cellular RNAs were extracted from stimulated and non-stimulated cells by TRIzol™ LS Reagent (Invitrogen, Carlsbad, CA, USA), according to the manufacturer's instructions. Adaptor-ligation PCR and microplate hybridization assays were carried out as previously described [10,17-21]. Briefly, 1 μg total RNA was converted to double-stranded cDNA using the SuperScript cDNA synthesis kit (Invitrogen), according to the manufacturer's instructions, except for priming with the BSL-18e primer adaptor that contained the *Sph*I site. The P20EA/10EA universal adaptors were ligated to the 5' end of BSL-18e-primed cDNA. This adaptor-ligated cDNA was cut with *Sph*I restriction enzyme, followed by removal of excess linkers and small cDNA fragments by polyethylene glycol precipitation. Next, three rounds of β-chain constant region (Cβ)-specific PCR were performed using the precipitated cDNA and Cβ sequence-specific oligonucleotide probes (SSOPs), to prepare amplified and biotinylated TCR cDNA pools. Hybridization was carried out between biotinylated PCR products TCRVβ SSOPs to detect amplified and biotinylated TCR cDNA immobilized on carboxylate-modified ELISA plates (Sumitomo bakelite, Tokyo, Japan). Hybridization was visualized with p-nitrophenylphosphate (Nacalai Tesque, Osaka, Japan). The visualized signals were estimated at 405 nm using Immunoreader NJ-2000 (Nihon Intermed, Tokyo, Japan). The relative expansion of the *TCRBV *region repertoire was calculated by the following formula: relative expansion of subfamily (%) = corresponding SSOP signal × 100/sum of total TCRVβ SSOP signals. The sequences of oligonucleotides used for amplified cDNA are shown in Table 1. We defined the increase as significant when: (i) the percentage was greater than the mean percentage +2 SD of five controls without SEC stimulation; and (ii) the actual percentage obtained in the assay was >10%.

**Table 1:** TCRBV-specific oligonucleotide probes

Name	Sequence 5'-3'	Target family
MVB1-1	ACGGTGCCCAGTCGTTTTAT	*BV1S1A1, 2*
MVB2-1	ACACGGGTCACTGATACGGA	*BV2S1*
MVB3-1	AGTGTCCTTCAAACTCACCTT	*BV3S1A1, 2*
MVB4-1	ATGGACAATCAGACTGCCTCA	*BV4S1*
MVB5-1	AGATAAAGGAAACCTGCCCAG	*BV5S1*
MVB5-2	GGATTCCTACCCAGCAGATTC	*BV2S2*
MVB6-1	GAAGGCTATGATGCGTCTCG	*BV6S1A1, 2*
MVB7-1	AAAGGATACAGGGTCTCACGG	*BV7S1*
MVB8-1	TGGCTTCCCTTTCTCAGACA	*BV8S1*
MVB8-2	GGCTACCCCCTCTCAGACAT	*BV8S2A1, 2, 3*
MVB8-3	CCAGAACAACGCAAGAAGACT	*BV8S3*
MVB9-1	TGAGAAGTTCCAATCCAGTCG	*BV9S1*
MVB10-1	TAAACGAAACAGTTCCAAGGC	*BV10S1A1, 2*
MVB11-1	ATAGATGATTCAGGGATGCCC	*BV11S1*
MVB12-1	CGCAGCAAGTCTCTTATGGAA	*BV12S1T*
MVB13-1	GCGACACAGCCACCTATCTC	*BV13S1*
MVB14-1	TTCATCCTAAGCACGGAGAAG	*BV14S1*
MVB15-1	TTCCCATCAGTCATCCCAAC	*BV15S1A1, 2*
MVB16-1	TCACTCTGAAAATCCAACCCA	*BV16S1A1, 2*
MVB17-1	GCATCCTGGAAATCCTATCCT	*BV17S1, 2P, 3*
MVB18-1	GGACAAGTTTCCAATCAGCCG	*BV18S1*
MVB19-1	AAAATGCCCTGCTAAGAAACC	*BV19S1*
MVB20-1	CAGCCTGGGAATCAGAACG	*BV20S1*

### Isolation and identification of bacterial strains on the skin surface

To evaluate the preferential bacterial colonization of the lesions, bacterial cultures were obtained from the facial skin surface of mice maintained under SPF and conventional conditions, with a sterile cotton swab-stick, inoculated onto salt egg yolk agar plates (Nissui, Tokyo, Japan), and incubated at 37°C. Ten colonies per mouse were picked at random and identified as subspecies of staphylococci, using an AN-ID Test-SP18 kit (Nissui), according to the manufacturer's instructions.

### Measurement of serum levels of antibody to peptidoglycan (PGN)

We quantitated IgG antibodies against PGN in DS-*Nh *and NC/Nga-*Nh *mouse sera in the following manner. One hundred microliters of PGN solution (10 μg/ml) in PBS (pH 7.6) was added to microtiter plate wells (Maxisorp™; Nunc, Roskilde, Denmark) and left to adsorb overnight at 4°C. The plates were then washed in PBS. The unbound sites on the plastic surface were blocked with 200 μl PBS containing 1% BSA, and left overnight at 4°C. The plates were washed twice with PBS and twice with PBS that contained 0.1% BSA. One hundred microliters of each serum sample, diluted 1:1000 in PBS that contained 0.1% BSA, was added to each well, and the plates were incubated overnight at 4°C. The plates were washed four times with PBS that contained 0.1% BSA, and 100 μl horseradish peroxidase (HRP)-conjugated anti-mouse IgG diluted in PBS that contained 0.1% BSA was added, and the plates were incubated at room temperature for 1 h. After four washes, 100 μl TMB substrate chromogen was added to each well. After 10–20 min at room temperature, the reaction was stopped with 100 μl 1 M HCl. The plates were read at 450 nm and values > 1.0 were considered positive.

### 2,4,6-trinitrochlorobenzene (TNCB) repeated-application dermatitis model

The TNCB repeated-application dermatitis model is popular and used as an artificial AD model. Shaved abdominal skin of mice was painted with 100 μl 5% TNCB dissolved in acetone/ethanol (1:4). Seven days after sensitization, the shaved dorsal skin was painted every other week with 100 μl 0.8% TNCB dissolved in olive oil. Clinical symptoms of individual mice treated with TNCB or vehicle were assessed for 5 weeks. Clinical skin condition was defined as cutaneous lesions that consisted of edema, erythema and erosion. These evaluation parameters were assessed by determining the total area of lesions on the shaved back. The scoring system was as follows: 0, not detectable; 1, <25% of total shaved skin surface; 2, <50% of total shaved skin surface; and 3, ≥ 50% of total shaved skin surface.

### Measurement of serum total IgE

Sera from mice before and after TNCB challenge were collected and stored at -80°C until use. Total IgE levels in sera were measured using an ELISA kit (Yamasa Shoyu Co., Ltd, Chiba, Japan).

### Statistical analysis

Statistical significance of differences was determined by Welch's *t *test, or one- or two-way analysis of variance.

## Results

### Generation of NC/Nga-*Nh *mice

NC/Nga-*Nh *mice were established by using ordinary breeding methods and segregated according to non-hair phenotype. NC/Nga and NC/Nga-*Nh *mice, as well as DS and DS-*Nh *mice, were genetically similar, except for the *Nh *locus, as shown in Table 2.

**Table 2:** Results of genetic monitoring using biochemical markers

	Number of mouse chromosome and genetic locus													
	
	1	1	1	2	3	4	4	5	6	7	7	8	8	9
mouse strains	*Idh1*	*Pep3*	*Akp1*	*Hc*	*Car2*	*Mup1*	*G6pd1*	*Pgm1*	*Ldr1*	*Gpi1*	*Hbb*	*Es1*	*Es2*	*Thy1*
DS	b	b	b	0	a	a	b	a	a	a	s	b	b	b
DS-*Nh*	b	b	b	0	a	a	b	a	a	a	s	b	b	b
NC/Nga	b	b	a	0	a	b	b	a	a	a	s	b	b	b
NC/Nga-*Nh*	b	b	a	0	a	b	b	a	a	a	s	b	b	b

														

	Number of mouse chromosome and genetic locus													
	
	9	9	11	17	17	6	6	11	12	14	14	17	17	19
mouse strains	*Mod1*	*Trf*	*Es3*	*H2K*	*H2D*	*Cd8a*	*Cd8b*	*Hba*	*IghC*	*Pnp*	*Es10*	*C3*	*Glo1*	*Cd5*

DS	a	b	c	s	-	a	a	c	a	a	a	b	a	b
DS-*Nh*	a	b	c	s	-	a	a	c	a	a	a	b	a	b
NC/Nga	a	b	c	d	-	b	b	c	a	a	a	a	a	b
NC/Nga-*Nh*	a	b	c	d	-	b	b	c	a	a	a	a	a	b

These tests were carried out by the ICLAS Monitoring Center, Kawasaki, Japan.

### Clinical and histological features in DS-*Nh *and NC/Nga-*Nh *mice

**Figure 1:**
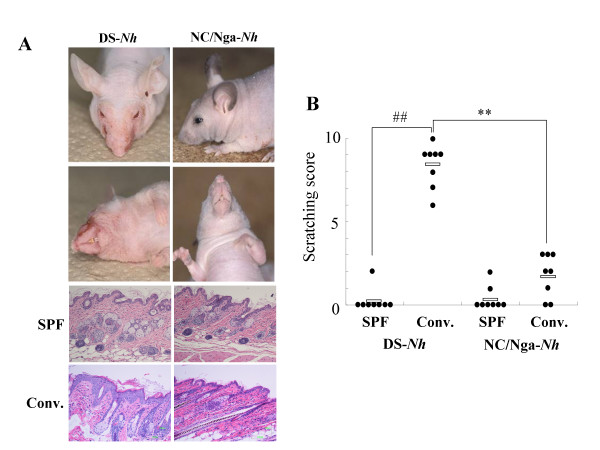
**Disease symptoms in DS-*Nh *and NC/Nga-*Nh *mice**. (A) Clinical features of DS-*Nh *and NC/Nga-*Nh *mice at 20 weeks of age kept under conventional or SPF conditions. (B) Evaluation of scratching and rubbing behavior in both strains at 20 weeks of age (*n *= 8) (SPF, kept under SPF conditions; Conv, kept under conventional conditions). White bar represents mean value of the grouped mice. (**, ##: significant differences at *p *< 0.01).

DS-*Nh *and NC/Nga-*Nh *mice displayed the hairless phenotype (Fig. 1A). We evaluated the clinical features and scratching behavior in DS-*Nh *and NC/Nga-*Nh *mice after 15 weeks under conventional conditions. Eczema was observed in the cheek and neck of DS-*Nh *mice, but not in NC/Nga-*Nh *mice. Inflammatory cell infiltration was observed in the skin of both mice, however hyperkeratosis was observed only in the skin of DS-*Nh *mice housed under conventional conditions (Fig. 1A). Inflammatory cell infiltration and hyperkeratosis were not observed in either strain housed under SPF conditions (Fig. 1A). Scratching and rubbing behavior was evaluated according to itching score. Scratching and rubbing behavior significantly increased in DS-*Nh *mice maintained under conventional conditions, but not in those maintained under SPF conditions, or in NC/Nga-*Nh *mice maintained under SPF or conventional conditions (Fig. 1B).

### TCR repertoire analysis

TCRVβ^b ^is the most common TCRVβ haplotype, and is found in the majority of laboratory mouse strains, including DS-*Nh *[11,22]. NC/Nga mice are characterized by the existence of a large central genomic deletion that removes several *TCRBV *gene segments [23]. Furthermore, we showed that SEC-producing *S. aureus *was cultured from the skin lesions of DS-*Nh *mice with AD, and that serum levels of anti-SEC antibodies were elevated. SEC plays an essential role in the development of AD in DS-*Nh *mice [10]. The *TCRBV *repertoire in spleen cells from DS-*Nh *and NC/Nga-*Nh *mice was analyzed to investigate the effects of deletion of *TCRBV *gene segments on TCRVβ selectivity for SEC.

**Figure 2:**
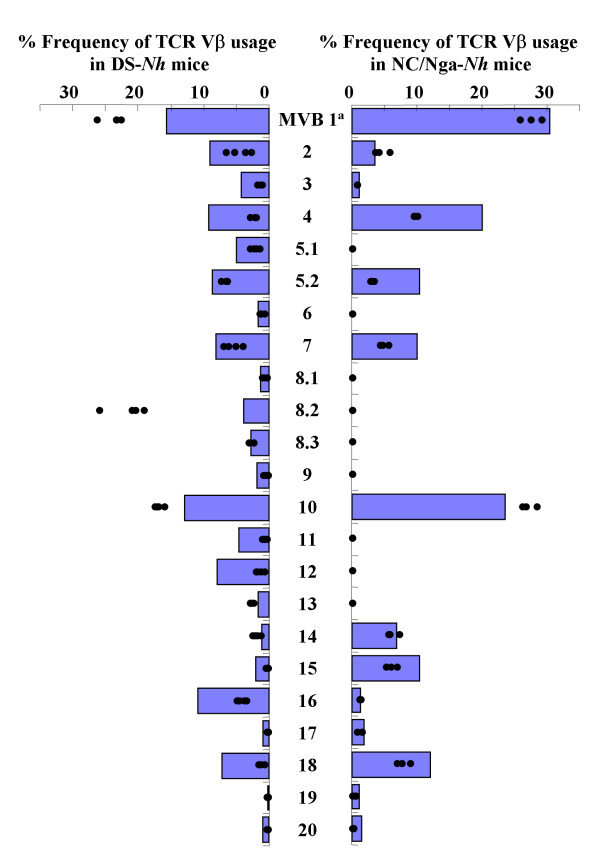
***TCRBV *repertoire analysis**. The *TCRBV* repertoires in spleen cells from five DS-*Nh* or NC/Nga-*Nh* mice without in vitro stimulation.Bar indicates mean +2 SD of the *TCRBV* repertoires in spleen cells from five DS-*Nh* or NC/Nga-*Nh* mice without *in vitro* stimulation. Each dot indicates the percentage frequency of *TCRBV*-bearing T cells in spleen cells stimulated *in vitro *with SEC.

Splenocytes from DS-*Nh *and NC/Nga-*Nh *mice were stimulated with SEC to investigate the *TCRBV *preference (Fig. 2). The *TCRBV *repertoire in spleen cells from DS-*Nh *mice was analyzed at day 4 after stimulation with and without SEC. The frequency of T cells bearing *TCRBV1*, *8S2 *and *10 *in all DS-*Nh *mice increased significantly at day 4 after SEC. The frequency of T cells bearing *TCRBV10 *in all NC/Nga-*Nh *mice increased significantly at day 4 after SEC.

### Serological analysis

We measured serum cytokine levels in DS-*Nh *and NC/Nga-*Nh *mice at 20 weeks of age. Only serum levels of G-CSF, MCP-1 and IL-1α were significantly increased in NC/Nga-*Nh *mice housed for 15 weeks under conventional conditions, compared with those at 20 weeks of age kept under SPF conditions. In DS-*Nh *mice housed for 15 weeks under conventional conditions, IL-12(p40), IL-12(p70), IL-13, IL-17, eotaxin, G-CSF, GM-CSF, IFN-γ, MCP-1, MIP-1α, IL-1α, IL-6, IL-9 and IL-10 were significantly increased, compared with those at 20 weeks of age kept under SPF conditions (Table 3).

**Table 3:** Cytokine levels in sera from NC/Nga-*Nh *and DS-*Nh *mice

	NC/Nga-*Nh *(SPF)		NC/Nga-*Nh *(Conv)		
	(pg/mL)	SD	(pg/mL)	SD	T-TEST
IL-12 (p40)	222.9	26.0	215.7	72.9	
IL-12 (p70)	314.9	76.2	317.7	105.7	
IL-13	30.2	5.4	29.2	9.3	
IL-17	309.4	162.9	478.8	213.9	
Eotaxin	1020.5	374.4	1248.4	117.9	
G-CSF	96.8	13.2	242.0	97.9	*
GM-CSF	51.2	12.7	63.8	4.9	
IFN-γ	35.3	6.4	41.5	8.2	
MCP-1	177.2	10.3	218.5	30.6	*
MIP-1α	1650.8	457.3	1926.4	213.7	
MIP-1β	62.6	15.0	113.6	100.1	
RANTES	345.0	207.4	437.4	63.8	
TNF-α	582.5	87.4	909.6	779.2	
IL-1α	40.5	4.8	70.7	22.4	*
IL-1β	37.9	12.1	43.3	57.2	
IL-6	16.6	3.1	50.8	47.2	
IL-9	397.0	76.5	358.8	73.5	
IL-10	94.3	21.7	89.8	49.1	
IL-18	193.2	285.1	205.2	249.3	

	DS-*Nh *(SPF)		DS-*Nh *(Conv)		
	(pg/mL)	SD	(pg/mL)	SD	T-TEST

IL-12 (p40)	201.5	64.4	396.3	115.9	*
IL-12 (p70)	260.4	109.2	1225.7	513.9	**
IL-13	26.3	6.5	66.8	14.5	**
IL-17	321.2	189.5	1078.3	275.1	**
Eotaxin	727.2	508.4	2257.0	733.5	**
G-CSF	77.0	27.3	1229.1	654.2	**
GM-CSF	43.4	11.3	120.6	25.3	**
IFN-γ	25.9	5.1	136.7	33.7	**
MCP-1	106.4	40.5	330.6	75.3	**
MIP-1α	1767.1	290.4	2738.2	656.8	*
MIP-1β	30.5	9.0	334.4	317.1	
RANTES	73.4	9.4	122.0	58.0	
TNF-α	497.4	64.4	2872.0	2350.3	
IL-1α	24.9	8.4	152.6	53.3	**
IL-1β	36.7	18.4	65.2	22.4	
IL-6	18.7	6.4	210.6	122.6	**
IL-9	307.4	49.4	657.1	162.4	**
IL-10	80.1	19.6	292.6	90.1	**
IL-18	369.2	56.3	263.5	37.9	**

KC, IL-2, IL-4 and IL-5: not detected.

SPF: kept under SPF conditions

Conv: kept under conventional conditions for 15 weeks

Data are expressed as means ± SD of five or six serum samples. [**: significant differences (*p *< 0.01), *: significant differences (*p *< 0.05)]

### Evaluation of bacterial colonization on the skin lesions

**Figure 3:**
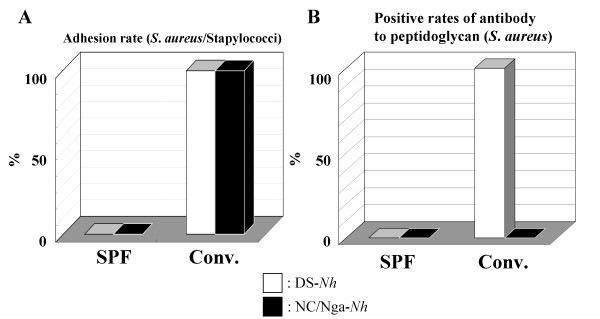
**Bacterial colonization of skin lesions**. (A) Isolation and identification of staphylococcal strains on the skin surface in both strains at 20 weeks of age (*n *= 5). (B) Measurement of serum levels of antibody to PGN in both strains at 20 weeks of age (*n *= 5).

To investigate the cause of the differences in serum cytokine profile between DS-*Nh *and NC/Nga-*Nh *mice, we evaluated the preferential bacterial colonization of the lesions. Although *S. aureus *was not isolated from either strain kept under SPF conditions, other bacterial species were completely replaced by *S. aureus *in both strains kept under conventional conditions for 15 weeks (Fig. 3A). PGN from *S. aureus *and TCRVβ haplotype have been reported recently to play an important role in IL-13 production [11]. We quantitated IgG antibodies against PGN in DS-*Nh *and NC/Nga-*Nh *mice sera, to investigate whether their immune systems were exposed to and activated by effectors derived from *S. aureus*. Antibodies against PGN were detected in DS-*Nh*, but not in NC/Nga-*Nh *mice (Fig. 3B).

### Repeated-hapten dermatitis model

**Figure 4:**
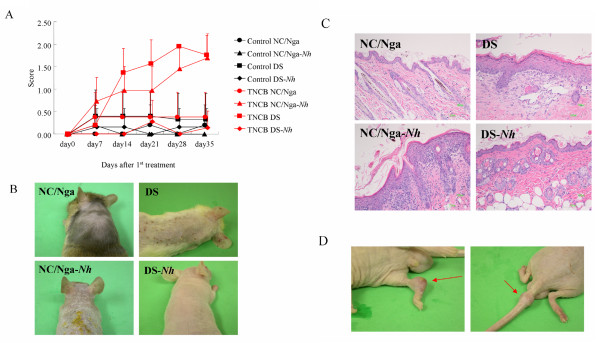
**Repeated application of TNCB in DS, DS-*Nh*, NC/Nga and NC/Nga-*Nh *mice**. (A) Evaluation of dermatitis in these mice. Each value represents mean ± SD of four or five mice. (B and C) Clinical features of skin in these mice. (D) Clinical features of joints in DS-*Nh *mice. Arrows indicate arthritis-like lesions.

Spontaneous dermatitis did not develop in NC/Nga-*Nh *mice kept under conventional conditions. Although spontaneous dermatitis models are more suitable than artificial ones to study human AD, it is difficult to construct spontaneous dermatitis models in mice. Thus, we evaluated DS, DS-*Nh*, NC/Nga and NC/Nga-*Nh *mice treated by repeated application of TNCB as a model of allergic contact dermatitis. Repeated-hapten dermatitis developed 3 weeks after the first sensitization in DS and NC/Nga-*Nh*, but not in DS-*Nh *and NC/Nga mice (Fig. 4A and 4B). Inflammatory cell infiltration and hyperkeratosis were observed in the skin of DS and NC/Nga-*Nh *mice (Fig. 4C). It was clear that Gly573Ser substitution in TRPV3 in NC/Nga-*Nh *mice significantly increased sensitivity to hapten compared with that in NC/Nga mice. On the other hand, we surprisingly found arthritis-like symptom in DS-*Nh *mice treated by repeated application of TNCB, despite the fact that dermatitis did not develop (Fig. 4D).

### Comparison of mast cell number and serum total IgE production

**Figure 5:**
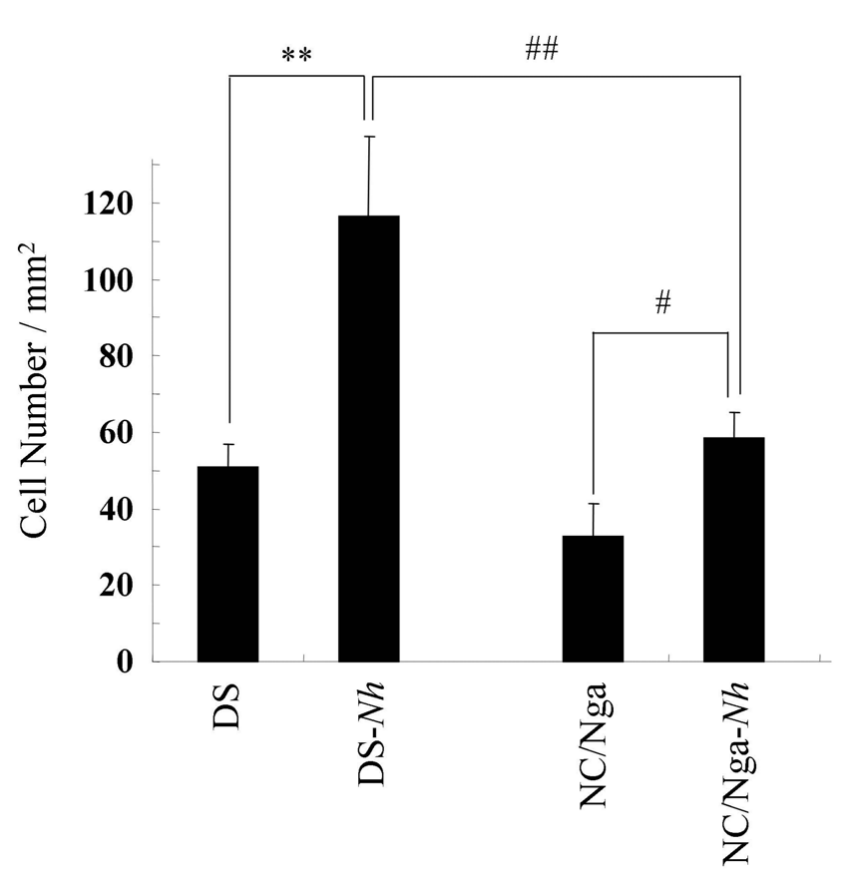
**Number of mast cells in skin from DS-*Nh*, NC/Nga-*Nh *and control mice**. Data represent the mean ± SD of six fields in six tissue samples. (**, ##: significant differences at *p *< 0.01), #: significant differences at *p *< 0.05).

**Figure 6:**
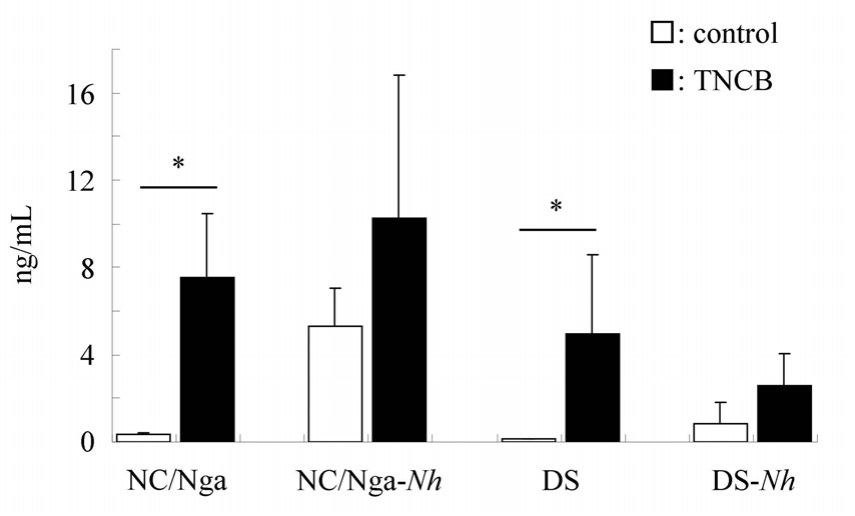
**Total serum IgE levels**. Data are expressed as means ± SD of four or five mice. (*: significant differences at *p *< 0.05).

To investigate the cause of differences in the development of spontaneous and artificial (repeated hapten) dermatitis, we counted the number of mast cells in the skin of five or six mice at 15 weeks of age, and measured serum total IgE levels. The number of mast cells in the skin of NC/Nga-*Nh *mice significantly increased compared with that in NC/Nga mice. The number of mast cells in the skin of DS-*Nh *mice significantly increased compared with that in DS and NC/Nga-*Nh *mice (Fig. 5). Although levels of serum total IgE were increased after TNCB application in these mice, serum IgE level in DS-*Nh *mice was lower than that measured in other strains. (Fig. 6).

## Discussion

We reported that TRPV3^Gly573Ser ^led to increased ion-channel activity in keratinocytes and caused spontaneous hairlessness and dermatitis in DS-*Nh *mice. These hairless and dermatitis phenotypes were both inherited in an autosomal dominant form and could not be segregated from each other. However, these phenotypes are segregated in C57BL/6-*Nh *mice and only the hairless phenotype is found [13]. This means that the penetrance of the TRPV3^Gly573Ser ^gene is different between hairless and dermatitis phenotypes. In the present study, we generated NC/Nga-*Nh *mice to investigate serologically and histologically the role of TRPV3^Gly573Ser ^in NC/Nga mice, which is a well-studied model of AD.

Environmental factors are major causes of AD. For example, in human AD, the Gram-positive bacterium *S. aureus *can be isolated from skin lesions and unaffected skin of >90% of patients, and it causes exacerbation of skin inflammation. In contrast, only 5% of normal subjects carry *S. aureus*, which is localized mainly in the nose and intertriginous areas of the skin. Moreover, anti-staphylococcal treatment is effective against exacerbation of eczema in AD [24]. These findings suggest that binding of *S. aureus *to the skin plays an important role in the development of AD. Transduction of the TRPV3^Gly573Ser ^gene into DS mice induces adhesion of SEC-producing *S. aureus *to the skin. Skin-adhered, SEC-producing *S. aureus *affect the host immune system, induce a shift to a Th2 immune response, and moreover, induce the development of AD [11]. On the other hand, Hashimoto *et al*. have reported recently the relationship between *S. aureus *and development of dermatitis in NC/Nga mice [16], and *S. aureus *adhered to the skin of NC/Nga-*Nh *mice in the present study. However, increased production of antibodies against PGN and development of dermatitis were not observed in NC/Nga-*Nh *mice. These results show clearly that adherence of *S. aureus *does not lead to activation of the immune system in NC/Nga-*Nh *mice. This discrepancy about the incidence of spontaneous dermatitis between laboratories may have been caused by superantigens derived from *S. aureus*. Thus, it is thought that the response between SEC and TCRVβ8S2 is important in the development of dermatitis because there are a lot of SEC-producing *S. aureus *in our laboratory [10]. However, NC/Nga-*Nh *and NC/Nga mice have a deficiency of a certain segment of the TCRVβ gene, including the TCRVβ8S2 gene. Our previous study has shown that TCRVβ haplotypes influence the incidence of spontaneous dermatitis [11]. Although C57BL/6-*Nh *mice appear to have more severe scratching behavior than NC/Nga-*Nh *mice, this may result from the difference in TCRVβ haplotypes and depend on mouse strain [13]. Interestingly, Habu *et al*. have reported recently that staphylococcal enterotoxin A (SEA) affects the immune system and induces a Th2 immune response [23]. These results suggest that SEA-producing *S. aureus *may play an important role in the development of dermatitis and scratching behavior in NC/Nga-*Nh *mice.

Although NC/Nga-*Nh *did not develop spontaneous dermatitis, the number of skin mast cells significantly increased compared with that in NC/Nga mice. Therefore, we compared hapten-repeated-application dermatitis, which is thought to be related closely to mast cells, in several mouse strains. There were significant increases in the number of mast cells in the skin of NC/Nga-*Nh *compared with NC/Nga mice. Previous data using mast-cell-deficient WBB6F1 W/WV mice have indicated that immediate contact hypersensitivity depends on an increase in dermal mast cells after repeated hapten application [25]. Furthermore, Matsukura *et al*. have reported that NC/Nga mice developed dermatitis by the application of TNCB after sensitization, which was carried out three times per week [26]. Hence, we hypothesized that the number of skin mast cells might be associated closely with hapten sensitivity in the skin. Mice with a certain number of mast cells did develop dermatitis by the milder application of TNCB after sensitization, which was carried out once per week. In the mice used in the present study, the number of mast cells in the skin had the following order: DS-*Nh *> DS ≈ NC/Nga-*Nh *> NC/Nga. In fact, DS and NC/Nga-*Nh *mice developed dermatitis after mild application of TNCB, and NC/Nga mice developed dermatitis after more severe treatment with TNCB (data not shown). Surprisingly, DS-*Nh *mice had the highest number of skin mast cells among these mice. However, DS-*Nh *mice did not develop dermatitis after the application of TNCB. Interestingly, we found that a certain type of arthritis developed as a result of this TNCB treatment in DS-*Nh *mice. We now speculate that this was caused partially by the different activation modes of mast cells. Our serological data indicate that the immune system in DS-*Nh *mice, but not that in NC/Nga-*Nh *mice, is exposed to superantigen-producing *S. aureus*. Also, a sufficient number of *S. aureus *did not adhere to DS mouse skin in our animal room. Furthermore, although levels of serum IgE increased after TNCB application in these mice, serum IgE level in DS-*Nh *mice was lower than that in other strains. According to these and other recent results [27], onset of arthritis by repeated application of TNCB in DS-*Nh *mice may be induced not by allergic responses between IgE and mast cells, but by inflammatory responses between superantigens and mast cells. Detailed analysis of repeated-hapten-induced arthritis is a topic for future study.

## Conclusion

In the present study, we bred the NC/Nga-*Nh *congenic strain to evaluate the contribution of TRPV3 to the development of AD in NC/Nga mice. The penetrance of the TRPV3^Gly573Ser ^gene for AD is not very high, although its penetrance for an increase in the number of skin mast cells is high. A gain-of-function mutation of TRPV3 as well as the number of mast cells and T cells with TCRVβ8S2 may be needed for the development of AD, and a high number of mast cells may play an important role in the development of arthritis. However, contrary to our expectations, spontaneous dermatitis did not develop in NC/Nga-*Nh *mice; they had a major response to hapten, and artificial dermatitis did develop. In conclusion, the model that uses NC/Nga-*Nh *mice contributes to better understanding of the pathophysiological mechanisms involved in the development of dermatitis.

## Competing interests

The authors declare that they have no competing interests.

## Authors' contributions

KI carried out the experimental work, analyzed the data and drafted the manuscript. TY carried out the experimental work, analyzed the data, and conceived the study and its design. TH participated in animal breeding and drafting of the manuscript. TS assisted in study design and helped to draft the manuscript. All authors have read and approved the final manuscript.
